# Internal stigmatization in patients with chronic migraine and medication overuse headache

**DOI:** 10.1186/s12883-024-03586-3

**Published:** 2024-03-19

**Authors:** Firdevs Ezgi Uçan Tokuç, Eylem Özaydın Göksu, Şennur Delibaş Katı

**Affiliations:** Republic of Turkey Ministry of Health Antalya Provincial Health Directorate, Neurology department, University of Health Sciences Antalya Training and Research Hospital, Antalya, Turkey

**Keywords:** Chronic migraine, Medication overuse headache, Ristsher’s stigmatization scale, Stigmatization, Alienation

## Abstract

**Objectives:**

Internalized stigma can have profound effects on how individuals with migraines and other primary headache disorders see themselves and their quality of life. We aimed to investigate internalized stigma in patients with chronic migraines and medication overuse headaches.

**Methods:**

A total of 57 patients (52 women, 5 men) were included in the study, 26 of these patients were affected by chronic migraine, 31 of them were affected by medication overuse headache and chronic migraine. The Internalized Stigma Scale in Mental Illness (Ristsher’s stigmatization scale) and General Health Questionnaire were applied to all patients.

**Results:**

In Ristsher’s stigmatization scale, which measures internalization of stigma, internalized stigmatization was more significant in patients with medication overuse headache than in patients with chronic migraine compared to groups (p:0.05). The subtitle of alienation was statistically significant when the groups were compared to all subscales in the form of alienation, confirmation of stereotypes, perceived discrimination, social withdrawal and resistance to stigma (p:0.05).

**Discussion:**

Although internal stigmatize has been observed in chronic migraine patients, medication overuse headache is also a type of headache with intense stigma. In addition, this internal stigma perhaps plays an active role in the transformation of chronic migraine patients to medication overuse headaches patient.

## Background

Stigma defined as negative labeling and stereotyping causes individuals to feel discredited, self-perception decreases, status loss and isolation of individuals from society for several reasons [1].

There are three types of stigmas: public, structural and internalized stigma. Public stigma refers to negative attitudes and stereotypes circulating in public. Structural stigma occurs when public stigma and the negative attitudes it entails are embedded in laws and organizational practices. The self-stigma occurs when individuals begin to assimilate and believe in negative assumptions about their stigmatized condition. While these forms of stigma contribute to the poor health outcomes of the individual, despite the clear importance of internalized stigma for health and psychological functionality, very few existing studies have considered directly internalized stigma and chronic pain [2–4]. It is known that the stigmatizing effects of some neurological diseases bring about deterioration of social relations and decreased quality of life and migraines are one of these diseases [5]. Migraines and other primary headache disorders are the second leading cause of disability in the world, according to The World Health Organization (WHO) [6].

According to ICDH 3 criteria, patients with headaches of 15 days or more in a month for at least 3 months and 8 of these headaches are migraine attacks are considered chronic migraine, while patients with primary headache and frequent use of painkillers, opoids or migraine attack medications (10 or 15 days/month, depending on medication) are considered MOH if their headaches become chronic and last more than 14 days [7].Only a few studies have examined stigma in relation to headaches, and almost all of them focus on migraines. However, it is thought that the stigma experienced by migraine patients greatly contributes to the disability [8]. Individuals with a stigmatized disease such as migraine internalize the negative characteristics attributed to the disease by society and cause this stigma to be applied to their perceptions and diseases. Internalized stigma can have profound effects on how individuals with migraines and other primary headache disorders see themselves and their quality of life. This concept has been demonstrated in patients with chronic migraines (CM), episodic migraines and epilepsy [1, 2]. However, it could not be demonstrated how much they internalized the negative characteristics related to the diseases of patients in CM and medication overuse headache patients (MOH). Based on this, we aimed to investigate internalized stigma in patients with chronic migraines and medication overuse headaches.

## Materials and methods

### Study design and patients

Patients who met the criteria according to ICDH3 for CM and for MOH + CM (developed MOH on the background of CM), between the ages of 18 and 65 who did not have any treatment for prophylaxis (especially SNRI) and no additional diseases and any drug therapy (especially antidepressants) were included in the study in our Antalya Education and Research Hospital headache outpatient clinic. The Internalized Stigma Scale in Mental Illness (ISSMI) and general health questionnaire were applied to patients.

The ISSMI scale is a Likert type scale which developed Ritsher and his colleagues, consisting of 29 substances that evaluate the internalized stigma. There are 5 subgroups: Alienation (6 items), Confirmation of stereotypes (7 items), Perceived discrimination (5 items), Social withdrawal (6 items), Resistance to stigma (5 items). The scale ranges from 4 to 91. High scores mean that internalized stigma of the person is more severe in a negative way [9]. General Health Questionnaire, on the other hand, determines the general psychopathology level and was developed by David Goldberg to catch acute mental disorders that are frequently encountered in community surveys. Each question examines symptoms over the past few weeks and has four options (“never happens, as usual, more often than usual, very often”) [10].

Patients answered the scales in a room within the Neurology Outpatient Clinic that would not be disturbed from the outside. The attending physician was around in case of any problem that may develop while the patient was filling out the scale. A level of education that could read and understand the scales with voluntary participation in the study was asked for. The Ethics Committee of the Antalya Education and Research Hospital approved this study (Number: 2020-023 KN: 3/26). All patients gave written consent for study participation.

### Statistical analysis

The statistical analysis was performed using the SPSS software, version 26. For normality test, Shapiro Wilk test was used. Mann-Whitney test and Kruskal Wallis non-parametric analysis of variances were used to determine the differences between the values calculated according to the groups. Relationships between variability were performed by using The Spearman’s Rank- Order Correlation analysis. Relationships between determined data groups were evaluated with T-Test and ANOVA test. *P* values less than 0.05 were considered statistically significant.

## Results

A total of 57 patients (52 women, 5 men; mean age ± sd; 36.6 ± 9.86 years) were included in the study, which was followed at the Antalya Education and Research Hospital Neurology headache clinic and met ICDH3 criteria. While 26 of these patients were CM patients, 31 of them were MOH + CM. 40.3% of the patients were primary school graduates, 31.6% were high school graduates, and 28.1% were university graduates. (Table 1). The most common medication used by MOH + CM patients was nonsteroidal anti-inflammatory drugs (NSAIDs) (64.5%), followed by triptans (22.5%). 12.9% patients used combination therapy (triptans and NSAIDs). In Ristsher’s stigmatization scale, which measures internalization of stigma, internalized stigmatization was more significant in patients with MOH + CM than in patients with CM compared to groups (p:0.05). When the groups were compared on all subscales including alienation, confirmation of stereotypes, perceived discrimination, social withdrawal and resistance to stigmatization, only the alienation subscale was statistically significant (p:0.05). No statistical difference was found between the two groups in terms of general health scale (p: 0.73) (Table 2).

**Table 1:** Sociodemographic Characteristics of the Participants

	Sociodemographic Characteristics of the Participants			
		MOH + CM (n:31)	CM (n:26)	
		N(%)	N(%)	
**Gender (Female)**		27(87%)	25(96.1%)	
**Education Level**				
Primary School		13(41.9%)	10(38.6%)	
High School		11(35.4%)	7(26.9%)	
University		7(22.5%)	9(34.6%)	
			Mean ± SD	Mean ± SD
**Age(years)**		39.1 ± 8.5	33.6 ± 10.6	
**Duration of Migraine(years)**		6.2 ± 2.8	5.5 ± 2.4	
**Duration of MOH(month)**		9 ± 3.4		

CM: chronic migraines, MOH: medication overuse headache

**Table 2:** Stigmatization Subgroups of the Paticipants

Stigmatization			
	MOH + CM(n:31) (Mean ± SD)	CM(n:26) (Mean ± SD)	*P*
Alienation	14.2 ± 4.7	11.9 ± 3.9	**0.05**
Approval of Stereotypes	13.6 ± 4.5	11.5 ± 3.9	0.06
Perceived Discrimination	8.4 ± 2.6	7.6 ± 2.5	0.06
Social Withdrawal	11.8 ± 4	10.1 ± 4.5	0.25
Resistance to Stigma	10.4 ± 2.1	9.3 ± 2.1	0.16
Stigmatization Total	58.5 ± 15.2	50.6 ± 14.5	**0.05**
General Health Scale	27.8 ± 8.3	27.8 ± 8.3	0.73

CM: chronic migraines, MOH: medication overuse headache

## Discussion

Our findings demonstrated that internalized stigma was higher in MOH + CM patients than in CM patients. In addition, in our study, the alienation scale was statistically significant in the MOH + CM patients compared to the CM patients. The Alienation subscale sought to measure the subjective experience of being less than a full member of society or having a ‘spoiled identity’ [9]. Previous studies have shown that Feelings of alienation are an important indicator of mental well-being. It has been associated with depressed mood, psychological distress, insomnia, and increased risk of suicide in some diseases. The valuable aspect of our study is that there are no studies investigating alienation in migraine patients [11].

The presence of stigma in neurological disease has been highly discussed, and there are currently a few studies on the presence of stigma among patients with migraine and tension-type headaches [12]. EFNA (European Federation of Neurological Associations) stated that 92% of patients with neurological diseases in 2020 were affected by stigma and 96% of the headache group was stigmatized [13].

Internalized stigma or self-stigma is the individual cognitive, emotional and behavioral effect caused by the internalization of negative traits added to individuals or diseases stigmatized by society. Internalized stigma of disease occurs when an individual experiencing imitated stigma or discriminatory social behavior towards their disease becomes aware of the negative cultural attitude towards the illness and in turn practices negative, stigmatized beliefs about themselves and their disease. There is evidence of internalized stigma among people with chronic and episodic migraines [2]. In the study conducted by Young, W B et al. in 2013, epilepsy patients and migraine patients were compared and it was demonstrated that CM patients felt more fluctuations and had a lower quality of life perception [1].

It has been reported that CM patients with internalized stigma have a lower quality of life and that the stigma experienced damages physician-patient relationships. Furthermore, studies on perceived stigma have shown that internalized stigma has an important role in treatment adherence and that better medication adherence is associated with lower stigma [14–16]. Individuals with a stigmatized disease such as migraine internalize the negative characteristics attributed to the disease by society and cause people to believe that they are responsible for the disease, causing shame and guilt in these patients. Therefore, patients try to hide their illness in such cases or avoid receiving medical attention [2].

A study conducted in the USA demonstrated that only about 10–15% of migraine patients refer to a neurologist or a specialist who deals with headaches. Patients who refuse to consult expert opinion prefer nonpharmacological treatments. The main reasons for this choice were thought to be stigmatization and official labeling, disclosure and therefore discriminatory treatment by the society. In addition, drug side effects, drug costs, dissatisfaction with available pharmacological treatment options, and lack of information about the benefits of preventive treatment also help shape these choices [16, 17].

Therefore, patients can quickly turn to analgesic use with the desire to relieve pain attacks, the fear of not being able to hide, explain and show that they are strong, and perhaps this drug abuse may be one of the most important underlying causes of headache [16–19].

There are some limitations in our study. Our patient numbers (especially man) were relatively low. Also, it would have been better if the patients of the two groups were compared in terms of psychological status by a valid questionnaire, because this issue can contribute to the development of MOH. A comparison could have been made with another headache type and this headache type plus MOH. These results could have clarified our thesis more clearly.

Nevertheless, no studies have been observed in the literature on the presence of stigma in patients with MOH + CM before. And our study suggested that MOH internal stigma is more dominant and our study is the first study in the literature suggesting the presence of MOH stigma.

## Conclusions

As a result, although internal stigmatize has been observed in CM patients, MOH is also a type of headache with intense stigma. In addition, this internal stigma perhaps plays an active role in the transformation of CM patients to MOH. In this regard, there is a need for prospective multicenter studies with large sample size.

## Data Availability

The datasets used and/or analysed during the current study are available from the corresponding author on reasonable request.
